# Editorial: Sociobiological interactions in brain health: from disparities to social epigenomics

**DOI:** 10.3389/fnagi.2024.1407486

**Published:** 2024-04-09

**Authors:** Hernando Santamaria-Garcia, Michael Corley, Agustin Ibañez

**Affiliations:** ^1^PhD Program of Neuroscience, Pontificia Universidad Javeriana, Bogotá, Colombia; ^2^Center for Memory and Cognition, Hospital Universitario San Ignacio, Bogotá, Colombia; ^3^Department of Medicine, Division of Infectious Diseases, Weill Cornell Medicine, New York, NY, United States; ^4^Latin American Brain Health Institute, Universidad Adolfo Ibañez, Santiago de Chile, Chile; ^5^Cognitive Neuroscience Center, Universidad de San Andrés, Buenos Aires, Argentina; ^6^Global Brain Health Institute (GBHI), University of California, San Francisco, San Francisco, CA, United States; ^7^Trinity College Dublin, Dublin, Ireland

**Keywords:** brain health, epigenetics, DNA methylation, biological clocks, genetics

Critical environmental factors like physical threats, air pollution, and infections, alongside social determinants of health (SDH) (Ibáñez et al., 2023) such as social disparities (Santamaria-Garcia et al., 2023), adversities, and psychosocial stress (Migeot and Ibáñez, 2023)—collectively referred to as the exposome—may alter molecules interacting with DNA, affecting gene activation or suppression (Ibanez and Zimmer, 2023). This control of gene expression, known as the epigenome, undergoes modifications due to the exposome, influencing regulatory mechanisms on genes associated with inflammation, metabolism, stress, neurotrophic factors, and neurodevelopment. Such changes are linked to the onset of psychiatric, neurological, and neurodegenerative disorders. Furthermore, epigenetic modifications in different genes can predict aging and affect brain and cognitive health.

Most research on the interplay between social, environmental, and biological factors in brain health lacks depth in exploring the genetics-epigenetics interplay, molecular mechanisms, biomarker impacts, and their clinical implications across neuropsychiatric disorders. Understanding social, genetic, and epigenetic interactions is crucial for comprehending disease etiology and risk. Against this backdrop, this Research Topic aims to gather studies that examine the intricate relationships between genetics, epigenetics, exposome factors, and their effects on molecular and neurocognitive processes in various neuropsychiatric disorders.

The first study of this issue investigated the genetic burden associated with Alzheimer's disease (AD) development and its clinical and neuroradiologic features. Suh et al. compared polygenic risk scores (PRS) with oligogenic risk scores (analyzing specific genes or single nucleotide polymorphisms) in predicting AD's clinical and neuroimaging markers. Using whole genome sequencing data from 1,545 individuals in the Alzheimer's Disease Neuroimaging Initiative (ADNI) cohort, the authors identified 20 genes correlated with key neuroimaging markers. These genes formed the basis of a new oligogenic score (adORS), which outperformed PRS in distinguishing between control subjects, those with mild cognitive impairment (MCI), and AD cases. The adORS identified genes such as ATF6, EFCAB11, ING5, SIK3, and CD46, noted in related research and other neurodegenerative diseases, underscoring adORS as a novel tool for differentiating clinical and neuroimaging features in AD and MCI.

The second study revealed that neurodegenerative diseases, such as amyotrophic lateral sclerosis (ALS), involve significant epigenetic changes that contribute to biological dysregulation and neurodegeneration despite well-known genetic patterns. Brusati et al. conducted an extensive DNA methylation analysis using an epigenome-wide association study on blood samples from 61 sALS patients and 61 healthy controls from an Italian cohort. The authors explored potential associations with chemical compounds and epigenetic drift (accumulation of stochastic epigenetic alterations), highlighting a pronounced increase in epigenetic drift and rare epivariations in sALS patients. Remarkably, this drift emphasized a group of genes within the neurotrophin signaling pathway. Moreover, they discovered rare epivariations associated with 153 genes, 88 of which have strong activity in the brain, marking the first identification of such variations in sALS patients. This study indicated the potential of epigenetic drift and epivariations as new diagnostic markers.

In the third study, Mareckova et al. explored the impact of epigenetic aging markers on cognitive brain health from prenatal stages to young adulthood. They found a stable epigenetic age gap (EpiAGE) from adolescence to the late twenties, with a modest correlation between higher EpiAGE, increased brain age gaps, and reduced intelligence scores in young adult women. This highlights the need for further research into the complex aging process and the relationship between epigenetic aging, brain development, and cognitive abilities.

The fourth study, conducted by Tian et al., delved into the interplay between lifestyle factors, such as alcohol consumption, and molecular pathways associated with AD through micro-RNA epigenetic regulation. Investigating the connection between AD, alcohol dependence, and ferroptosis, the study identified key genes. It explored how micro-RNA processes mediate the effects of these gene pathways on AD and alcohol consumption relationships. Through immune infiltration, functional enrichment analysis, machine learning, and consensus clustering, they pinpointed CYBB, STEAP3, and ACSL4 as crucial genes and elucidated their roles in the interaction between AD and alcohol consumption. CYBB mutations were linked to impaired phagocyte functions, leading to increased infections, inflammation, and various diseases. STEAP3, associated with ferroptosis, regulates diseases via immune pathways and is influenced by m^∧^6A modifications affecting oxygen metabolism. Acyl-CoA Synthetase Long-Chain Family Member 4 (ACSL4) plays a vital role in the metabolism of polyunsaturated fatty acids, impacting microglial inflammation and ferroptosis sensitivity, relevant to Parkinson's disease and AD. This advanced analysis revealed connections between risk behaviors, genetic predispositions, gene expression, and molecular pathways regulating cell death in AD, opening new research and potential therapeutic strategy avenues.

Together, the studies in this Research Topic highlight the importance of advancing studies that reveal the complex interactions between genetic factors, epigenetic mechanisms, and the exposome. Moreover, we must enhance our understanding of how these interactions could mechanistically explain changes in biological and molecular pathways associated with psychiatric and neurological disorders and neurodegeneration. Additionally, more precise knowledge of these mechanistic biological changes and their impacts on neurocognitive processes could increase the role of new genetic-epigenetic measures for biological aging detection, disease diagnosis, monitoring, and prognosis. Moreover, broader knowledge and use of genetic-epigenetic measures in clinical settings could open new avenues for disease risk factor detection, therapeutic development, and brain health promotion.

## Author contributions

HS-G: Writing – review & editing, Writing – original draft, Conceptualization. MC: Writing – review & editing, Writing – original draft, Conceptualization. AI: Writing – review & editing, Writing – original draft, Conceptualization.
